# Topical insecticide treatments to protect dogs from sand fly vectors of leishmaniasis.

**DOI:** 10.3201/eid0705.017516

**Published:** 2001

**Authors:** R Reithinger, U Teodoro, C R Davies

**Affiliations:** Disease Control & Vector Biology, Infectious & Tropical Diseases, London School of Hygiene &Tropical Medicine, United Kingdom. rreithinger@yahoo.co.uk

## Abstract

We compared the susceptibility of sand fly vectors to four topical insecticide treatments applied to domestic dogs, a reservoir of human leishmaniasis. Dogs were exposed to sand flies pretreatment and at 1 week, 1 month, and 2 months posttreatment. Sand fly bloodfeeding and survival rate of both fed and unfed flies were significantly reduced by the permethrin, deltamethrin, and fenthion treatments, but diazinon had no effect. The survival rate of bloodfed sand flies was reduced by up to 86% with deltamethrin collars. The antifeeding effect suggests that deltamethrin collars may be recommended to dog owners to protect their pets from sandfly- borne diseases. The combined effects on sand fly feeding and survival indicate that epidemiologic, community-based trials are warranted to test whether deltamethrin collars could reduce the incidence of canine and, hence, human leishmaniasis.


### Topical Insecticide Treatments to Protect Dogs from Sand Fly Vectors of Leishmaniasis

**Richard Reithinger,*† Ueslei Teodoro,† and Clive R. Davies***
*London School of Hygiene & Tropical Medicine, London, United Kingdom; and †Universidade Estatal de Maringá, Maringá, Paraná, Brazil

We compared the susceptibility of sand fly vectors to four topical insecticide treatments applied to domestic dogs, a reservoir of human leishmaniasis. Dogs were exposed to sand flies pretreatment and at 1 week, 1 month, and 2 months posttreatment. Sand fly bloodfeeding and survival rate of both fed and unfed flies were significantly reduced by the permethrin, deltamethrin, and fenthion treatments, but diazinon had no effect. The survival rate of bloodfed sand flies was reduced by up to 86% with deltamethrin collars. The antifeeding effect suggests that deltamethrin collars may be recommended to dog owners to protect their pets from sandfly-borne diseases. The combined effects on sand fly feeding and survival indicate that epidemiologic, community-based trials are warranted to test whether deltamethrin collars could reduce the incidence of canine and, hence, human leishmaniasis.

Leishmaniases are a group of zoonotic diseases transmitted to humans and animals by the bite of phlebotomine sand flies (1). Worldwide, the leishmaniases are the third most important vector-borne disease (after malaria and sleeping sickness), accounting for an estimated 1.98 million disability-adjusted life-years and 57,000 deaths annually (2). In the past 20 years, the number of human leishmaniasis cases has dramatically increased, a trend that shows no signs of abating (3). Much of this increase may result from adaptation of *Leishmania* transmission cycles to the peridomestic environment as a response to deforestation and urbanization (4). Another explanation is that the leishmaniases are a common opportunistic infection in HIV-infected persons (5).

In Latin America, as in North Africa and Asia, *Leishmania* infections in dogs have great public health importance, as dogs are the reservoir hosts of zoonotic visceral leishmaniasis (ZVL) caused by *Leishmania infantum (=Le. chagasi*). Dogs are also the suggested reservoir hosts of American cutaneous leishmaniasis (ACL) caused by *Le. (Viannia)* spp. (6) and may have a role in the transmission cycle of cutaneous leishmaniasis in the Old World caused by *Le. tropica* (7). Canine leishmaniasis is mainly a veterinary problem in Europe (where estimates suggest that up to 7 million dogs are at risk for infection [8]) and the United States (where there has been a recent widely publicized outbreak in foxhounds in New York [9]).

In addition to treating patients and spraying houses with insecticide, ZVL control programs (notably in Brazil and China) often target the reservoir hosts by culling infected dogs. A similar culling policy has been suggested for controlling ACL where domestic transmission has been demonstrated. However, the impact of dog-culling programs on (human and canine) ZVL incidence has been questioned on theoretical and practical grounds (10,11), and results of controlled intervention trials are contradictory (6,12-14). Treating infected dogs with antileishmanial drugs (e.g., pentavalent antimonials, amphotericin B, aminosidine, or allopurinol) is not a practical control policy, not only because of the prohibitive cost involved but also because of high relapse rates (up to 74% [15]) among treated and clinically cured dogs. Moreover, a high proportion of clinically cured dogs remain parasitologically positive and therefore infectious to the sand fly vector (16).

While awaiting a leishmanial vaccine (17), alternative canine leishmaniasis control strategies are urgently needed. One proposal has been to cut transmission by treating domestic dogs with insecticides (18)–lotions or insecticide-impregnated dog collars (1) originally developed for flea and tick control (19). Our work directly compares the anti-feeding and lethal effects of deltamethrin-impregnated (DM) dog collars with those of alternative topical insecticide applications. The study also increases the number of sand fly species against which insecticide-impregnated dog collars have now been tested and is the first such study to test their impact on a vector of ACL (*Lu. intermedia*), which is known to feed on both humans and dogs (20).

### Materials and Methods

### Study Site and Protocol

All 17 dogs used in the experimental trial came from the Fazenda Palmital, a large farm 40 km from Maringá, Estado do Paraná, Brazil (23°40'S, 52°25'W). Mongrel dogs were stationed individually inside cages (50-cm width x 60-cm height x 60-cm depth) and were exposed overnight (±22.30 to 5.30 hours, i.e., for 7 hours) to an average 96 (69 to 121) wild-caught sand flies introduced through sleeves of closed net curtain tents (100 cm x 180 cm x 130 cm). Sand flies had been caught the same night by mouth aspirator inside open chicken pens at the Fazenda Marista (21.00 to 22.00 hours),

Address for correspondence: Richard Reithinger, Disease Control & Vector Biology, Infectious & Tropical Diseases, London School of Hygiene & Tropical Medicine, Keppel Street, London WC1E 7HT, United Kingdom; fax: 44-20-7467-9536; e-mail: rreithinger@yahoo.co.uk a neighboring farm. Previous studies have shown that >85% of all collections at the Fazenda Marista are *Lu. intermedia* (Teodoro et al., unpub. data), which was confirmed by microscopically identifying to species a subset of caught sand flies: Of 200 unfed and 40 bloodfed sand flies, 172 (86%) and 35 (88%) were identified as *Lu. intermedia,* respectively; the remainder were *Lu. whitmani.* Sand flies were collected from the tents the following morning by using a mouth aspirator, placed into suspended gauze cubic cages, and maintained on sucrose solution for a further 17 hours at 24°C to 26°C and 90% to 95% relative humidity. After 24 hours, flies were identified by sex and scored as either dead or alive, and bloodfed or unfed.

All dogs were exposed to sand flies before treatment (day 0) and again at 5 to 12 days posttreatment (dpt), 32 to 36 dpt, and 58 to 65 dpt. Three time points were compared: 1 week, 1 month, and 2 months. Four treatments were compared: 1) 40 mg/g DM-impregnated collars (Scalibor, Intervet International GmbH, Wiesbaden, Germany) (n = 5); 2) 15% diazinon-impregnated collars (Canovel, Pfizer, United Kingdom) (n = 3); 3) application of 1 mL of 0.65 g/mL permethrin topical lotion (Pulvex, Coopers Brasil Ltda, São Paulo, Brazil) (n = 3); and 4) application of 1 mL of 15% fenthion topical lotion (Pulfim, Bayer S.A. Brasil, Barueri, Brazil) (n = 3). Collars were attached around the necks of the dogs. Topical lotions were applied directly onto the skin after the dog's hair was separated at the nape of the neck. Three untreated dogs (negative controls) were exposed to sand flies at the same time points to adjust for any background changes in sand fly feeding and survival rates over time. Changes in climatic conditions (temperature and relative humidity) were measured at the start and end of each bioassay.

### Data Analysis

The effects of the different treatments (in relation to the negative control) at each time point were tested by using General Linear Models (21) in the computer package STATA, i.e., by analyses of deviance, specifying binomial errors, of the log odds that the sand fly bloodfeeding rate was diminished as a result of treatment or that the sand fly death rate was increased as a result of treatment. Any significant overdispersion was corrected by rescaling the model using the ratio of the residual deviance to residual degrees of freedom. Maximal models incorporated the effects of dog age and size and climatic conditions along with treatment. The significance of each variable was tested by back-step analysis of deviance, i.e., by observing whether these variables explained a significant ($p < 0.05$) proportion of the deviance remaining after their removal from the model. Variables were excluded from the models in order of least significance until only significant variables were retained in the minimum adequate model.

### Results

An average of 49 (19 to 86) female and 22 (2 to 47) male sand flies were recovered from the tents the following morning (mean recovery rate 74%). The sex ratio was remarkably constant throughout the experiment, with no significant differences detected with time or treatment (median proportion of females recovered: 0.68; 25% to 75% quartiles: 0.61 to 0.75). Sand fly bloodfeeding and death rates were unaffected by dog age and size or climatic conditions throughout the trial. No dogs had visible side effects from the different treatments.

### Sand Fly Bloodfeeding Rate

In the absence of treatment, the average sand fly bloodfeeding rate was 42% (Table 1) (Figure). There was no significant difference between the bloodfeeding rate on negative control dogs and the rates on any of the four treatment groups before treatment ($p > 0.2$ for all four comparisons). Neither were there any significant differences in bloodfeeding rates on treated and untreated dogs at the first time point (1 week), although there was some suggestion of a reduction on the dogs treated with permethrin ($p = 0.088$) and DM ($p = 0.083$). Bloodfeeding rates were significantly lower on dogs treated with DM, permethrin, and fenthion (compared with untreated dogs) at both 1 month ($p < 0.001$; $p = 0.010$; and $p = 0.005$, respectively) and 2 months ($p < 0.001$; $p = 0.004$; and $p = 0.018$, respectively). At both time points, the reduction in bloodfeeding rate was greatest on DM-treated dogs (Table 2), although the difference with permethrin- and fenthion-treated dogs could not be demonstrated statistically. The greatest antifeeding effect in all trials was detected on DM-treated dogs after 2 months, when bloodfeeding rates were reduced by 69% (95% confidence intervals [CI] 43,78). A similar pattern was demonstrated when bloodfeeding rates posttreatment were compared with bloodfeeding rates pretreatment on the same dogs (rather than with bloodfeeding rates on control dogs at the same time points). In these analyses, a significant reduction in bloodfeeding was detected on DM-treated dogs at all three time points: at 1 week ($p = 0.012$), 1 month ($p < 0.001$), and 2 months ($p < 0.001$). In contrast, bloodfeeding rates only dropped significantly by 1 month on fenthion-treated dogs and by 2 months on permethrin-treated dogs. No significant reduction in bloodfeeding was detected on diazinon-treated dogs at any time point, when compared with either negative control dogs or pretreatment controls.

### Sand Fly Death

In the absence of treatment, the average sand fly death rate of unfed and bloodfed sand flies was 12% and 2%, respectively (Table 1) (Figure). There was no significant difference between the death rate of bloodfed sand flies on negative control dogs and the equivalent rates on any of the four treatment groups before treatment ($p > 0.4$ for all four comparisons). In comparison with untreated dogs, deaths of bloodfed flies was significantly increased at 1 week by 11%, i.e., 5.7-fold (95% CI 1.1, 20.5) as a result of permethrin treatment ($p = 0.037$) and at 1 month by 33%, i.e., 27-fold (4.4, 66) by fenthion treatment ($p = 0.001$) and by 30%, i.e., 25-fold (4.2, 63) by DM treatment ($p = 0.001$). No significant treatment effects on deaths of bloodfed sand flies were detected at 2 months.

Before treatment, there was no significant difference between the death rate of unfed sand flies on negative control dogs and the equivalent rates on three of the treatment groups ($p > 0.4$ for all three comparisons), but deaths of unfed sand flies in the group allocated to have DM treatment were significantly less ($p = 0.020$) than for the control group. The effect of this minor pretreatment bias was to make it harder to detect any significant increase in deaths as a result of DM treatment. With this caveat, we were unable to detect any

Table 1. Experimental dog trial comparing effects of topical insecticide applications on feeding and survival rates of female sand flies[a]

| Dog | TM | 0 dpt | | 5-12 dpt | | 32-36 dpt | | 58-54 dpt | |
|---|---|---|---|---|---|---|---|---|---|
| | | B | U | B | U | B | U | B | U |
| 1 | C | 40 (1) | 37 (3) | 37 (0) | 38 (6) | 25 (0) | 34 (4) | 23 (1) | 30 (4) |
| 2 | C | 43 (1) | 34 (8) | 28 (0) | 27 (2) | 26 (1) | 39 (6) | 25 (1) | 33 (3) |
| 3 | C | 31 (1) | 44 (5) | 24 (2) | 40 (7) | 30 (0) | 29 (2) | 14 (3) | 37 (8) |
| 4 | DM | 20 (1) | 28 (2) | 21 (2) | 31(10) | 14 (3) | 34(14) | 8 (1) | 36(11) |
| 5 | DM | 24 (0) | 54 (4) | 16 (1) | 27 (5) | 7 (3) | 37 (8) | ND[b] | ND[b] |
| 6 | DM | 47 (1) | 39 (2) | 9 (1) | 53 (7) | 8 (2) | 37(14) | 5 (1) | 43 (9) |
| 7 | DM | 26 (0) | 28 (3) | 3 (0) | 20(10) | 4 (2) | 31(20) | 4 (2) | 34(15) |
| 8 | DM | 25 (1) | 34 (1) | 14 (0) | 26 (5) | 6 (2) | 26(23) | 5 (1) | 23(19) |
| 9 | DZ | 38 (0) | 40 (5) | 32 (0) | 20 (3) | 23 (1) | 18 (6) | ND[c] | ND[c] |
| 10 | DZ | 26 (0) | 38 (4) | 36 (2) | 8 (4) | 29 (1) | 27 (7) | ND[c] | ND[c] |
| 11 | DZ | 16 (0) | 34 (5) | 15 (1) | 21 (2) | 13 (0) | 39 (6) | 17 (0) | 29 (4) |
| 12 | PM | 17 (0) | 27 (6) | 6 (2) | 35 (7) | 9 (1) | 21 (4) | 8 (1) | 23 (8) |
| 13 | PM | 16 (1) | 39 (3) | 10 (1) | 24(11) | 21 (1) | 38(19) | 11 (0) | 38(11) |
| 14 | PM | 22 (0) | 14 (3) | 15 (1) | 31 (9) | 13 (2) | 36(10) | 10 (1) | 39 (9) |
| 15 | F | 22 (1) | 47 (7) | 15 (2) | 15 (6) | 6 (0) | 17 (9) | 9 (1) | 28(13) |
| 16 | F | 29 (1) | 22 (8) | 27 (1) | 15(13) | 11 (4) | 28(19) | 16 (0) | 37 (7) |
| 17 | F | 10 (1) | 21 (2) | 16 (0) | 44 (5) | 4 (3) | 15(10) | 8 (3) | 33 (8) |

[a] Dead sand flies (after 24 hrs) are in parentheses.
[b] Dog 5 was killed by his owner
[c] Dogs 9 and 10 moved.
Abbreviations: B, bloodfed sand flies; C, control; DM, deltamethrin-impregnated dog collar; dpt, days posttreatment; DZ, diazinon-impregnated dog collar; F, fenthion topical lotion; PM, permethrin topical lotion; ND, not done; TM, treatment; U, unfed sand flies.

significant differences in the death rate of unfed sand flies at 1 week. However, in comparison with untreated dogs, death of unfed sand flies was significantly increased by 41%, i.e., 4.1-fold (1.7 to 6.6) at 1 month by DM treatment (p = 0.004) and by 58%, i.e., 5.4-fold (2.3 to 7.6) by fenthion treatment (p = 0.001). The death rate of unfed sand flies at 2 months was significantly increased by 29%, i.e., 2.6-fold (1.02 to 4.7) by DM treatment (p = 0.046).

Finally, we analyzed the combined effects of treatment on bloodfeeding and the deaths of bloodfed flies, i.e., how treatment affected the proportion of females that both took a bloodmeal and survived 24 hours. As expected, the diazinon collar had no effect at any time point. None of the treatments had a significant effect at 1 week (although the effects of DM and permethrin were of borderline significance: p = 0.064 and p = 0.053, respectively). At 1 month, DM (p <0.001), permethrin (p = 0.003), and fenthion (p <0.001) all caused a significant reduction, but the effect of DM was significantly greater than that of permethrin (p = 0.001). A significant reduction was again detected at 2 months for DM (p <0.001), permethrin (p = 0.008), and fenthion (p = 0.015); and the effect of DM was significantly greater than that of both permethrin (p = 0.019) and fenthion (p = 0.01).

### Discussion

The observed reduction in *Lu. intermedia* bloodfeeding and increase in sand fly deaths support the hypothesis that topical insecticides, including collars, could protect dogs against leishmaniasis. Bloodfeeding rates of sand flies were reduced from 1 month posttreatment not only on dogs with collars impregnated with deltamethrin but also on dogs treated with topical lotions of permethrin and fenthion. No effect was detected for dogs with diazinon collars. Although no significant difference between the antifeeding effects of DM, permethrin, and fenthion was detected, the reduction due to DM was greater than that due to either permethrin or fenthion at both 1 month and 2 months after treatment (Table 2). The failure to detect any significant effect on bloodfeeding 1 week after treatment is probably due to the time required for the lipophilic insecticides to spread in the dermal secretions over the dog's body (22). The manufacturers currently recommend that the DM collar be put on approximately 2 weeks before an anticipated sand fly challenge. However, a significant fivefold increase in death rates was detected for bloodfed sand flies on permethrin-treated dogs after 1 week, suggesting that topical application of permethrin lotion can have a relatively immediate effect. This effect is relatively short-lived, however, as no lethal effect on bloodfed sand flies was detected at 1 month or 2 months posttreatment, and no effect on unfed flies was detected at any time point. In contrast, topical fenthion application showed greater persistence, significantly increasing death rates of both unfed and bloodfed flies at 1 month after treatment. The lethal effect of the DM collar was the most persistent treatment, with significant effects on bloodfed flies after 1 month and on unfed flies at both 1 month and 2 months posttreatment. The failure to detect a significant impact on bloodfed flies after 2 months may be due to the relatively few bloodfed flies at that time point (due to the high antifeeding effect). The diazinon-treated collar did not affect the death rate of either bloodfed or unfed flies at any time.

The first evidence that topical insecticides could be used to

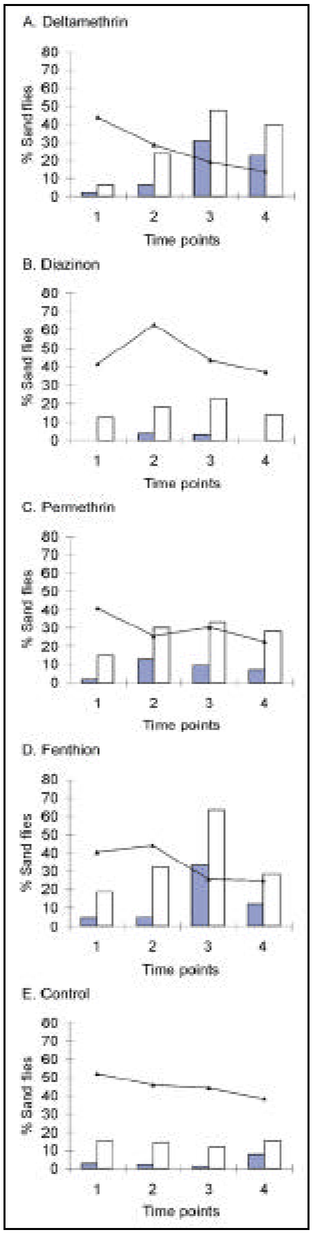

Figure. Comparison of various topical insecticide applications to protect dogs from sand fly bites. Represented are percentage bloodfed (line), dead bloodfed (dark columns), and dead unfed (white columns) sand flies. Time point (TP) 1: 0 days posttreatment (dpt); TP2: 5-12 dpt; TP3: 32-35 dpt and TP4: 58-65 dpt.

control ZVL came from experimental studies in China. After it was demonstrated that DM bath treatment reduced the bloodfeeding rate and survival rate of *Phlebotomus chinensis* sand flies exposed to an unnatural host (hamsters) (23), researchers found that the bloodfeeding rates of *P. chinensis* exposed to dogs for 8 hours were significantly reduced (from 62% to 4%) by dipping dogs in 25 ppm DM and that none of the sand flies (unfed or fed) exposed to treated dogs survived (18); this effect persisted for up to 104 days. Field evidence for the impact of topical insecticides comes from a community-based trial in China, where ZVL transmission was apparently interrupted after 2 years of treatment (two rounds per year) of all village dogs in 50 ppm DM baths (24). However, the trial results must be interpreted with caution, as no control villages were included in the study.

In contrast to insecticide lotions, the effects of DM collars on sand fly bloodfeeding and survival persisted for up to 8 months under experimental conditions in France using colonized *P. perniciosus* (25). Once collars had been applied for 2 weeks, blood-feeding consistently dropped by 90% and death rates increased by 51% during the experiment. When similar trials were carried out in Spain (26), the antifeeding effect dropped significantly from >90% during the first 4 months to 84% after 6 months, and the lethal effect dropped steadily from 76% after 2 weeks to 42% after 6 months. Both trials demonstrate that, when collars are used, the effects persist much longer than when dogs are dipped in DM. The potentially wide applicability of DM collars for protecting dogs against sand flies has since been demonstrated by experimental trials with *P. papatasi* in Iran (27).

Comparisons of the results of our trial with those of similar studies with DM collars (25-27) are difficult because of differences in experimental protocols. For example, we used wild-caught *Lu. intermedia*, whereas Killick-Kendrick et al. (25) and Lucientes (26) used 7- to 15-day-old, colonized *P. perniciosus*. This may account for the relatively low lethal effect detected in our trial, as old and colonized flies tend to be more sensitive to low doses of insecticide. Second, whereas dogs were exposed to high sand fly densities (mean 155 female flies per dog recovered after 2 hours) in the study by Killick-Kendrick et al. (25), our dogs were exposed to low fly densities (mean 49 female flies per dog recovered after 7 hours). The longer exposure time in our experiments, which was chosen to increase sand fly bloodfeeding rates and contact time with the various treatments, may have contributed to the decreased sand fly recovery rates we observed. Third, in contrast to all previously reported studies, we chose not to sedate the dogs to make the trial conditions as natural as possible. This provides a further reason for the slightly lower recovery rates of sand flies after exposure, compared with previous studies.

The potential protection against sand fly-transmitted diseases afforded by collars to individual dogs depends solely on their antifeeding effect. Although trials measuring impact on dog infection rates are still required, the entomologic results we report strongly indicate that DM collars may be recommended to dog owners to protect their dogs from leishmaniasis. This could include dog owners traveling to leishmaniasis-endemic countries, such as in southern Europe, as imported leishmaniasis cases of dogs with a travel history to Mediterranean leishmaniasis-endemic areas are increasingly common (15,28). With the recent change in the United Kingdom quarantine laws, this is likely to be of increasing concern to British dog owners.

The putative epidemiologic impact of DM collars on leishmaniasis transmission will depend on reducing not only the number of sand flies feeding on dogs but also the survival of those flies that do feed so that they are less likely to transmit *Leishmania* when taking a subsequent bloodmeal on a susceptible dog or human. Combining the effects on the bloodfeeding and death rates, we calculate that DM collars reduced the number of bloodfed flies that survive 24 hours by an average of 91% at 1 month posttreatment and by 81% at 2 months posttreatment. This was greater than the reductions of 61% (not significant) and 37% ($p = 0.010$) for

Table 2. Percentage reduction in sand fly bloodfeeding after application of topical insecticide

| | Percent reduction in sand fly bloodfeeding (95% CI) | | | | | |
|---|---|---|---|---|---|---|
| TM | 5-12 dpt | | 32-36 dpt | | 58-65 dpt | |
| DM | 37.6 | (5.3, 68.0) | 56.7 | (38.7, 70.5) | 68.5 | (42.8, 77.6) |
| DZ | -37 | (-77.1, 13.3) | 1.4 | (-23.1, 24.7) | 3.4 | (-40.1, 40.0) |
| PM | 44.1 | (-7.2, 76.2) | 31.6 | (7.8, 51.4) | 49.2 | (14.3, 61.6) |
| F | 4.2 | (44.8, 48.3) | 41.4 | (13.3, 62.9) | 43.1 | (53.0, 56.0) |

Abbreviations: CI, confidence intervals; DM, deltamethrin-impregnated dog collar; dpt, days post treatment; DZ, diazinon-impregnated dog collar; F, fenthion topical lotion; PM, permethrin topical lotion; TM, treatment.

fenthion treatment, and 37% ($p = 0.001$) and 41% ($p = 0.019$) for permethrin treatment, at 1 month and 2 months, respectively. Thus, there appears to be a clear advantage in terms of effectiveness in using DM collars versus the two topical lotions. Another advantage of collars is that their presence on dogs, when following up treated dogs during a control campaign, is proof that the insecticide is applied. Balancing these advantages, pour-on lotions are probably easier to use, and wear and tear is not a concern. Further studies are clearly needed to decide the optimal mechanisms of insecticide application and delivery (topical lotions, sprays, powders, dips, collars, or ear tags) before widespread implementation can be recommended.

In conclusion, the entomologic results reported here are sufficiently encouraging to warrant the undertaking of trials measuring the epidemiologic impact of communitywide DM collar implementation. Trials are required in a variety of endemic ZVL zones, as the impact of targeting domestic dogs in a given zone will be determined by the relative importance of sylvatic canids or other mammals as alternative reservoir hosts. Where topical insecticide applications are shown to be effective in intervention trials, the choice of whether to use topical lotions or insecticide-impregnated dog collars as a public health tool will ultimately depend on 1) the relative strength and persistence of their effects on sand fly blood-feeding and survival; 2) the cost of the intervention; and 3) the practical applicability of these tools in the field (e.g., the willingness of the community to apply the topical formulation to their dogs or the efficiency with which they replace collars that have detached). The implementation of any topical insecticide treatment (including collars) is more likely to have the consent of a population at risk than the highly unpopular dog culling policy that is currently practiced.

### Acknowledgments

We thank Mycon Luiz Prina and the Companhia Melhoramentos Norte de Paraná (Brazil) for logistical support; Pfizer (United Kingdom) and Intervet International GmbH (Germany) for supplying the collars used in the study; and Paul Coleman and Diarmid Campbell-Lendrum for comments on the manuscript.

This study was funded by the Sir Halley Stewart Trust and the LSHTM Sir Patrick Manson Bequest Fund.

Mr. Reithinger is a doctoral student at the London School of of Hygiene & Tropical Medicine. He has been working on leishmaniasis epidemiology research projects in South America. His research interests include molecular diagnostics, epidemiology, immunology, control, and economics of infectious diseases.

### References

1. Ashford RW. Leishmaniasis reservoirs and their significance in control. Clin Dermatol 1996;14:523-32.
2. World Health Organization. The world health report 2000. Health systems: improving performance. Geneva: The Organization; 2000. Also available at URL: http://www.who.int/htr
3. Desjeux P. Leishmaniasis. Public health aspects and control. Clin Dermatol 1996;14:417-23.
4. Arias JR, Monteiro PS, Zicker F. The reemergence of visceral leishmaniasis in Brazil. Emerg Infect Dis 1996;2:145-6.
5. Alvar J, Canavate C, Gutierrez-Solar B, Jimenez M, Laguna F, Lopez-Velez R, et al. *Leishmania* and human immunodeficiency virus coinfection: the first ten years. Clin Microbiol Rev 1997;10:298-319.
6. Reithinger R, Davies CR. Is the domestic dog (*Canis familiaris*) a reservoir host of American cutaneous leishmaniasis? A critical review of the current evidence. Am J Trop Med Hyg 1999;61:530-41.
7. Dereure J, Rioux JA, Gallego M, Perieres J, Pratlong F, Mahjour J, et al. *Leishmania tropica* in Morocco: infection in dogs. Trans R Soc Trop Med Hyg 1991;85:595.
8. Gradoni L. Epizootiology of canine leishmaniasis in Southern Europe. In: Killick-Kendrick R, editor. Canine leishmaniasis: an update. Wiesbaden: Hoechst Roussel Vet; 1999. p. 32-9.
9. Rooney J, Schantz PM, Jackson J, Breitschwerdt E, Steurer F, et al. Visceral leishmaniasis in the United States—a multistate outbreak in dogs. Am J Trop Med Hyg 2000;62 Suppl:269.
10. Tesh RB. Control of zoonotic visceral leishmaniasis: is it time to change strategies? Am J Trop Med Hyg 1995;52:287-92.
11. Dye C. The logic of visceral leishmaniasis control. Am J Trop Med Hyg 1996;55:125-30.
12. Dietze R, Barros GA, Teixeira L, Harris J, Michelson K, Falqueto A, et al. Effect of eliminating seropositive canines on the transmission of visceral leishmaniasis in Brazil. Clin Infect Dis 1997;25:1240-2.
13. Ashford DA, David JR, Freire M, David R, Sherlock I, Eulalio MC, et al. Studies on the control of visceral leishmaniasis: impact of dog control on canine and human visceral leishmaniasis in Jacobina, Brazil. Am J Trop Med Hyg 1998;59:53-7.
14. Vieira JB, Coelho GE. Leishmaniose visceral o calazar: aspectos epidemiologicos e de controle. Rev Soc Bras Med Trop 1998;31 Suppl II:85-92.
15. Slappendael RJ, Teske E. The effect of intravenous or subcutaneous administration of meglumine antimonate (Glucantime) in dogs with leishmaniasis. A randomized clinical trial. Vet Q 1997;19:10-3.
16. Reithinger R. Control of leishmaniasis. Vet Rec 1999;144:732.
17. Modabber F. First generation leishmaniasis vaccines in clinical development: moving, but what next. Current Opinion in Anti-Infective Investigational Drugs 2000;2:35-9.
18. Guanghua X, Changfa J, Xinzhong C, Zyhongwei S, Yumei H. Deltamethrin bath of domestic dog in the prevention of sand fly bite. Endemic Disease Bulletin 1994;9:32-4.
19. Marsella R. Advances in flea control. Vet Clin North Am Small Anim Pract 1999;29:1407-24.
20. Campbell-Lendrum DH, Pinto MC, Brandão-Filho SP, De Souza AA, Ready PD, Davies CR. Experimental comparison of anthropophily between geographically dispersed populations of *Lutzomyia whitmani* (Diptera: Psychodidae). Med Vet Entomol 1999;13:299-309.
21. Crawley MJ. GLIM for ecologists. Oxford: Blackwell Science; 1993.
22. Miller TA, Salgado VL. The mode of action of pyrethroids on insects. In: Leahey JP, editor. The pyrethroid insecticides. London: Taylor & Francis; 1985. p. 43-97.
23. Changfa J, Guanghua X, Yumei H, Zhongwei S. Studies on the effect of deltamethrin bath treatment of hamsters infected with *Leishmania donovani* for interrupting kala-azar transmission. Chinese Journal of Parasitology and Parasitic Diseases 1994;12:300-2.
24. Guanghua X, Changfa J, Yumei H, Zyhongwei S, Peize X, Weikun X, et al. Studies on the deltamethrin-medicated bath of domestic dogs for interrupting visceral leishmaniasis transmission. Chinese Journal of Parasitology and Parasitic Diseases 1995;13:178-81.
25. Killick-Kendrick R, Killick-Kendrick M, Focheux C, Dereure J, Puech M-P, Cadiègues MC. Protection of dogs from bites of phlebotomine sand flies by deltamethrin collars for control of canine leishmaniasis. Med Vet Entomol 1997;11:15-111.
26. Lucientes J. Laboratory observations on the protection of dogs from the bites of *Phlebotomus perniciosus* with Scalibor protector bands: preliminary results. In: Killick-Kendrick R, editor. Canine leishmaniasis: an update. Wiesbaden: Hoechst Roussel Vet; 1999. p. 92-4.
27. Halbig P, Hodjati MH, Mazloumi-Gavgani AS, Mohite H, Davies CR. Further evidence that deltamethrin-impregnated collars protect domestic dogs from sand fly bites. Med Vet Entomol 2000;14:223-6.
28. Gothe R, Nolte I, Kraft W. Leishmaniasis in dogs in Germany: epidemiological case analysis and alternatives to conventional causal therapy. Tierärtzliche Praxis 1997;25:68-73.
